# Anatomy-Weighted CTDIvol from Routine CT Metadata: A Patient-Specific, Multi-Vendor Study Using Deep-Learning Segmentation

**DOI:** 10.3390/tomography12090125

**Published:** 2026-08-30

**Authors:** Shuji Yamamoto

**Affiliations:** Institute of One, LISIT Co., Ltd., Tokyo 150-0044, Japan; yamamoto@lisit.jp

**Keywords:** computed tomography, CTDIvol, tube-current modulation, deep-learning segmentation, TotalSegmentator, image-based dosimetry indices, reproducibility, open data

## Abstract

**Background/Objectives:** The volume computed tomography (CT) dose index (CTDIvol) is a scanner output, not an organ dose, and cannot express how tube-current modulation varies along a patient. An organ-specific weighted CTDIvol addressing this has been reported before, in single-institution cohorts and often from inputs routine archives do not retain. New here is not the quantity but what an open, multi-vendor operationalisation reveals: whether its inputs survive archive curation and what the fallback costs when they do not. **Methods:** Forty abdominal CT series, ten per manufacturer, were drawn from the Cancer Imaging Archive and twelve organs segmented with TotalSegmentator at inference. Of 480 requested organ–series combinations, 455 were produced. A rule-based acquisition-constancy criterion admitted 39 series. **Results:** Modulation weights spanned 0.59 to 1.69, so the index departs from the whole-scan CTDIvol by up to 70% within one acquisition. A recorded CTDIvol survived in 29 of 40 archived headers and was reconstructable in 5 and unavailable in 6, availability differing markedly between manufacturers. Forcing that reconstruction on series that did retain a value agreed to within 12% on three scanner models and diverged by 58% and 84% on two others. Estimated organ mass was broadly consistent with International Commission on Radiological Protection (ICRP) Publication 89 for liver and kidneys. **Conclusions:** This index is not an absorbed dose; the implementation is open.

## Simple Summary

CT scanners report a single dose number for the whole scan, yet the dose is delivered unevenly across organs. We built an open, freely available software pipeline that uses deep-learning segmentation to outline each abdominal organ on routine CT images and then derives a patient-specific, organ-level dose *index* directly from data the scanner already records. Across scans from four manufacturers, we found that this organ-level information can be recovered for some scanners but is entirely absent for others because the values it needs were not kept in the archived scan records. All software, results, and data provenance are fully open, so the work can be reproduced and extended.

## 1. Introduction

Two different things are routinely conflated when computed tomography (CT) dose is discussed. The first is that a *dose index* is not a *dose*: the volume CT dose index (CTDIvol) describes the output of a scanner into a standard cylinder of acrylic and is a property of the acquisition rather than of the patient in it—a distinction set out explicitly by McCollough et al. [1]. The second is that a single value per series cannot express variation along the patient: almost every modern acquisition modulates the tube current longitudinally [2], so the conditions over the liver and over the bladder are not the same, and one number for the series conceals that.

That second point is well established. Khatonabadi et al. demonstrated that a *regional* or *organ-specific* CTDIvol, formed from the modulation profile over an organ’s own location, tracks Monte Carlo organ dose far better than the whole-scan value, raising the coefficient of determination for liver dose from 0.26 to 0.86 [3]. Tian et al. formalised a weighted organ-specific CTDIvol computed from the modulation profile and used it, with organ–dose coefficients, to predict organ dose prospectively [4].

The quantity examined here is therefore not new, and no new index is proposed. This study takes the organ-specific weighted CTDIvol already reported in the literature and asks a different question: what happens when it is operationalised openly, end to end, on heterogeneous archived data from four manufacturers using automated segmentation and nothing but the metadata a scanner already writes?

Existing work only partially answers that question. Longitudinal dose indices, DICOM-header-derived modulation profiles, and TotalSegmentator-assisted dose calculations have each been investigated, but in different settings and for different endpoints. Li et al. characterised the size-specific dose estimate as a function of longitudinal position, SSDE(z), under both fixed and modulated tube current [5]. Nuntue et al. derived tube-current-modulation profiles from DICOM headers and used them, with Monte Carlo simulation and physical measurement, to improve absorbed organ dose estimates in abdominal CT [6]. Eom et al. incorporated TotalSegmentator into an automated effective-dose calculation on clinical PET/CT [7]. What remains insufficiently characterised is whether the organ-weighting layer can be reconstructed end to end from heterogeneous archived DICOM alone, how often the required inputs survive archive curation and de-identification, how acquisition–parameter constancy can be verified, and how the resulting quantity behaves across manufacturers when patient-specific contours are obtained automatically. Deep-learning segmentation is what makes the attempt practical at scale: a general-purpose segmenter such as TotalSegmentator [8], built on nnU-Net [9], produces abdominal organ masks from a routine series in seconds at inference only, so the anatomy is now effectively free.

This study is accordingly an open, multi-vendor operationalisation and empirical characterisation of a previously reported quantity: the whole-scan CTDIvol scaled by a dimensionless, organ-specific weight formed from the recorded per-slice tube current over that organ’s segmented longitudinal extent, referred to here as the anatomy-weighted CTDIvol index. It is explicitly not an estimate of absorbed organ dose, and Section 2.11 sets out what it does not account for.

Converting an index of this kind into an absorbed organ dose in milligray requires CTDIvol-normalised organ–dose coefficients, computed by Monte Carlo simulation over anthropomorphic patient models and corrected for patient size [4]. Such coefficient sets exist, are well validated, and are in routine use; the index reported here does not replace them and is not offered as a surrogate for their output.

The contributions are stated below in terms of what is computed and by what rule since that is where the novelty of an operationalisation lies:

1. **An end-to-end computation from archived metadata alone.** Per-slice tube current I(z) is read from (0018,1151) on every image; the series is resampled onto a uniform slice grid. Twelve organ masks are obtained from a general-purpose segmenter at inference; each organ’s longitudinal extent is taken from the extreme slices of its own mask, and the organ weight is the mean of I(z) over that extent divided by the mean over the whole series. The index is weight times the whole-scan CTDIvol. Nothing in the chain requires projection data, a manual contour, or a value the scanner did not already write.
2. **A multi-vendor empirical characterisation of the resulting quantity**—its range, its within-acquisition spread, and its behaviour across four manufacturers—is computed by the same code on all series so that between-vendor differences cannot arise from between-site processing.
3. **A measurement of how often the inputs survive archive curation and of what happens when one of them does not.** The measurement is a direct inspection of the archived headers of all 40 series for the two attributes the index needs: per-slice tube current and a whole-scan CTDIvol in (0018, 9345). Where the second is absent, it can sometimes be rebuilt from acquisition physics, and Section 3.5 reports how far that reconstruction agrees with the recorded value on the series where both can be obtained. What is new is the pairing: retention rates measured on a multi-vendor archive sample, together with the accuracy and the coverage of the fallback that the gaps force a retrospective study onto.
4. **A rule-based acquisition-constancy criterion** that makes the proportionality assumption behind the weighting testable rather than implicit: a series is admitted only if every attribute that governs scanner output other than tube current is constant within it to a tolerance justified in Section 2.9 from the resolution at which those attributes are stored.
5. **An external reference comparison of attenuation-derived estimated organ mass against International Commission on Radiological Protection (ICRP) Publication 89 values [10].** This is not a separate study but the only external check available to the pipeline: no ground-truth organ mass exists for archived series, and organ mass is the one intermediate quantity the pipeline produces that can be compared with a published reference at all. Agreement bounds how far the segmentation and the Hounsfield-to-density mapping can jointly be wrong, which is what makes the weights downstream of them worth reporting.

## 2. Materials and Methods

### 2.1. Data Selection Without Bulk Download

**Figure 1:**
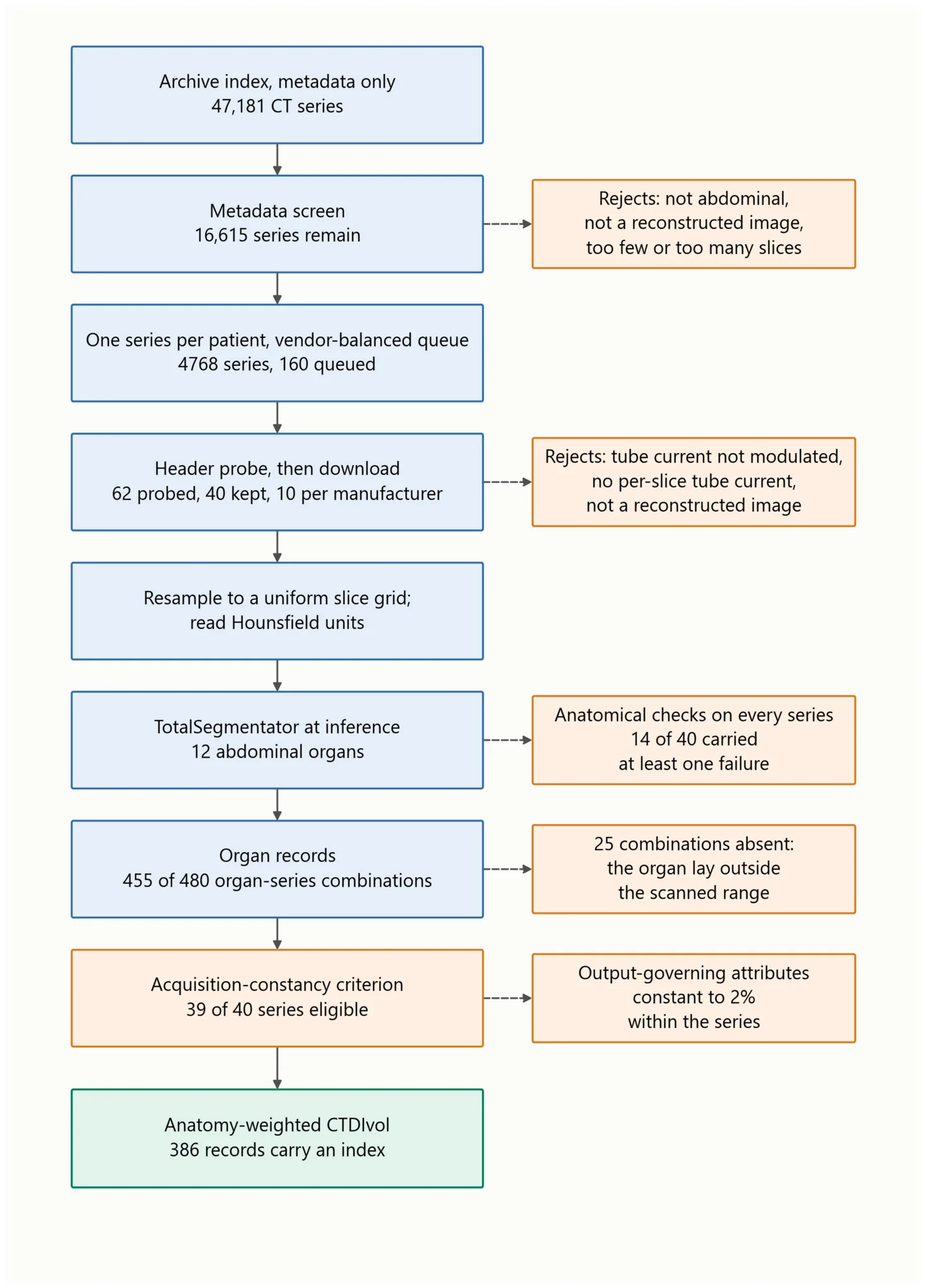
Data formation and verification. Blue: stages that produce or transform data, with the number of series or records carried forward. Orange: verification, each box naming what its stage rejects or flags. Green: the reported quantity. Every count is read from the shipped result files.

Handing a collection manifest to a bulk downloader fetches an entire collection, which for the low-dose CT collection is of the order of 600 GB, most of it raw projection data irrelevant to this work. We therefore used a metadata-first procedure over the public NBIA REST API with four stages—index, screen, probe, and download—in which only the last transfers a series. Figure 1 sets out the whole pipeline: the stages of data formation, the number of series surviving each, and the verification attached to each stage together with what it rejects.

All series in this study were public, de-identified data from the Cancer Imaging Archive, used under the individual collection licences recorded in the provenance file that accompanies the software. The archive’s submission process removes protected health information from both headers and pixel data before publication while retaining the attributes research requires [11], and no imaging was redistributed by this work: series were identified by Series Instance UID, collection, and licence so that any of them could be re-fetched. The study required no ethical approval since it used only such data and enrolled no participants.

All imaging was drawn from the Cancer Imaging Archive [12]. The candidate index was built from 47,181 CT series across 21 abdominal collections, read as series-level JSON with no pixel data. Candidates were seeded from the public-archive survey distributed with the companion software release [13]—a software record, not a peer-reviewed study—and supplemented by direct collection queries. A metadata screen then rejected the following, in order and with each rejection counted: non-patient collections (imaging phantoms and de-identification benchmarks); projection and raw-data series, of which 398 were refused at this stage; non-diagnostic series (localisers, dose reports, and screen captures); series shorter than 40 or longer than 1200 images; and series outside the abdomen. One series was retained per patient per collection, and each manufacturer’s quota was drawn round-robin across its collections so that manufacturer was not confounded with a single collection.

Ten series per manufacturer were chosen for what the study measures. Every quantity reported here was a per-series or per-organ property computed by identical code, so the comparison that mattered was between organs within a patient, where each series was its own control and 40 series yielded 455 organ records. Ten per manufacturer supported a median and an interquartile range for a manufacturer, which is what is reported, and did not support a distributional claim about one, which is not. Nothing in the design was powered by adding series: a larger cohort would narrow those interquartile ranges without changing what the index is or whether its inputs survive archive curation.

The selection was not a random sample of clinical CT and cannot be treated as one. Three stages shaped it. The metadata screen kept abdominal, diagnostic, reconstructed series of moderate length, so unusual acquisitions were removed by construction. The probe required per-slice tube current recorded on every probed image and genuine modulation, which excluded fixed-current protocols entirely and, as Section 3.3 shows, correlates with manufacturer through what the archive retained. The collections themselves are oncological, so body habitus and organ appearance were those of a cancer population rather than of a screening one. The direction of each effect can be stated even though its size cannot: the cohort was biased towards modern modulated abdominal protocols on scanners whose archived headers were complete, which was the population in which an anatomy-weighted index was computable at all, and the availability fractions in Section 3.3 were therefore an upper bound on what a less selective cohort would yield.

### 2.2. Inclusion Criteria and Header Probing

Sixty-two surviving candidates were probed by fetching six image headers each and judged on four requirements: per-slice tube current present on every probed slice; that current genuinely modulated (peak-to-peak over mean at least 0.02, so header rounding is not mistaken for modulation); a defined Hounsfield rescale; and a reconstructed-image SOP class with at least 40 slices spanning at least 120 mm. Forty series were kept, ten per manufacturer.

No imaging was redistributed. Each retained series was identified in the shipped provenance record by collection, collection DOI, Series Instance UID, manufacturer, model, licence, and retrieval date. All forty series were retrieved under Creative Commons Attribution licences—33 under CC BY 4.0 and 7 under CC BY 3.0, as recorded per series in data/PROVENANCE.json; users must in every case observe the licence terms of the originating collection and the TCIA Data Usage Policy.

### 2.3. Resolving the Slice Grid

Organ volume is a voxel count multiplied by a voxel volume, so slice spacing multiplies every volume and every mass estimate. Two properties of archived series make the obvious calculation wrong, and both are silent.

First, the file count is not always the position count. One series in this cohort contained 160 images at 119 distinct longitudinal positions; taking the spacing as the extent divided by the number of images gave 3.71 mm, where the true spacing was 5.0 mm, and stacking the duplicated images repeated anatomy so that organs occupied more slices than they physically do. We therefore resolved the grid explicitly: one image per position, spacing from the median step between neighbouring positions, and a uniformity check that refused a series whose steps varied by more than 2%. Two series proved to be a pair of reconstructions interleaved under a single Series Instance UID; for these, the largest regular sub-grid was taken, accepted only when it preserved the full longitudinal extent.

Second, the ordering of the slice axis was not guaranteed. Slice Location (0020, 1041) ran opposite in sign to Image Position (Patient) on three of the four manufacturers in this sample, so a series sorted by the former arrived head-first. The segmentation was unaffected because the geometry handed to the segmenter was built from patient coordinates, but the array index ceased to mean “towards the head”, which reversed every reported organ extent. Volumes were therefore canonicalised so that index zero was the most inferior slice, with the tube current reordered alongside since *I(z)* was paired to the slice axis by index.

### 2.4. Reading Hounsfield Units

Outside the reconstruction circle an image carries a padding value rather than a measurement, and that value must be replaced with air before anything is measured. Pixel Padding Value (0028, 0120) has a value representation that depends on Pixel Representation, and this is not reliably honoured: in this sample, one export writes 63,536 with an unsigned representation on signed pixel data, which is the two’s complement encoding of the −2000 intended. Read literally, that places the padding threshold above every Hounsfield value in the image, and the volume becomes uniform air. Four series were affected, with no symptom other than a segmenter returning empty masks. We reinterpreted the padding value against Pixel Representation and additionally refused any padding rule that would blank essentially the whole image.

### 2.5. Segmentation

Twelve abdominal organs were segmented with TotalSegmentator v2.17 [8], total task, 1.5 mm full-resolution model at inference only; no weights were trained, modified, or redistributed.

TotalSegmentator is an nnU-Net model [9]. nnU-Net is not a fixed architecture but a self-configuring pipeline: from the spacing, size, and intensity distribution of a training set, it derives the patch size, the batch size, the pooling depth, and the normalisation scheme, and it instantiates an encoder–decoder convolutional network of the U-Net family with those settings. The self-configuration and its validation are examined further in [14]. The network used here was the three-dimensional full-resolution variant, which processed overlapping patches of the volume and aggregated them with Gaussian weighting so that a voxel near a patch border was decided mainly by the patch in which it sat centrally. The published model was trained on a corpus of computed tomography covering 104 anatomical structures across a wide range of scanners, protocols, and body regions [8]; the twelve abdominal structures used here were a subset of its output classes. Inference used the released weights with the default configuration on one GPU in a separate child process because nnU-Net spawned its own worker processes and doing so from a long-lived parent leaked them on Windows.

The series was written to NIfTI by our own code, with an affine constructed from the DICOM patient coordinates, and the masks returned on that same grid; the correspondence between mask voxel and image voxel was therefore the identity by construction and was asserted rather than assumed. A mirrored segmentation would otherwise produce entirely plausible volumes and Hounsfield values while pairing every organ with the wrong anatomy.

**Figure 2:**
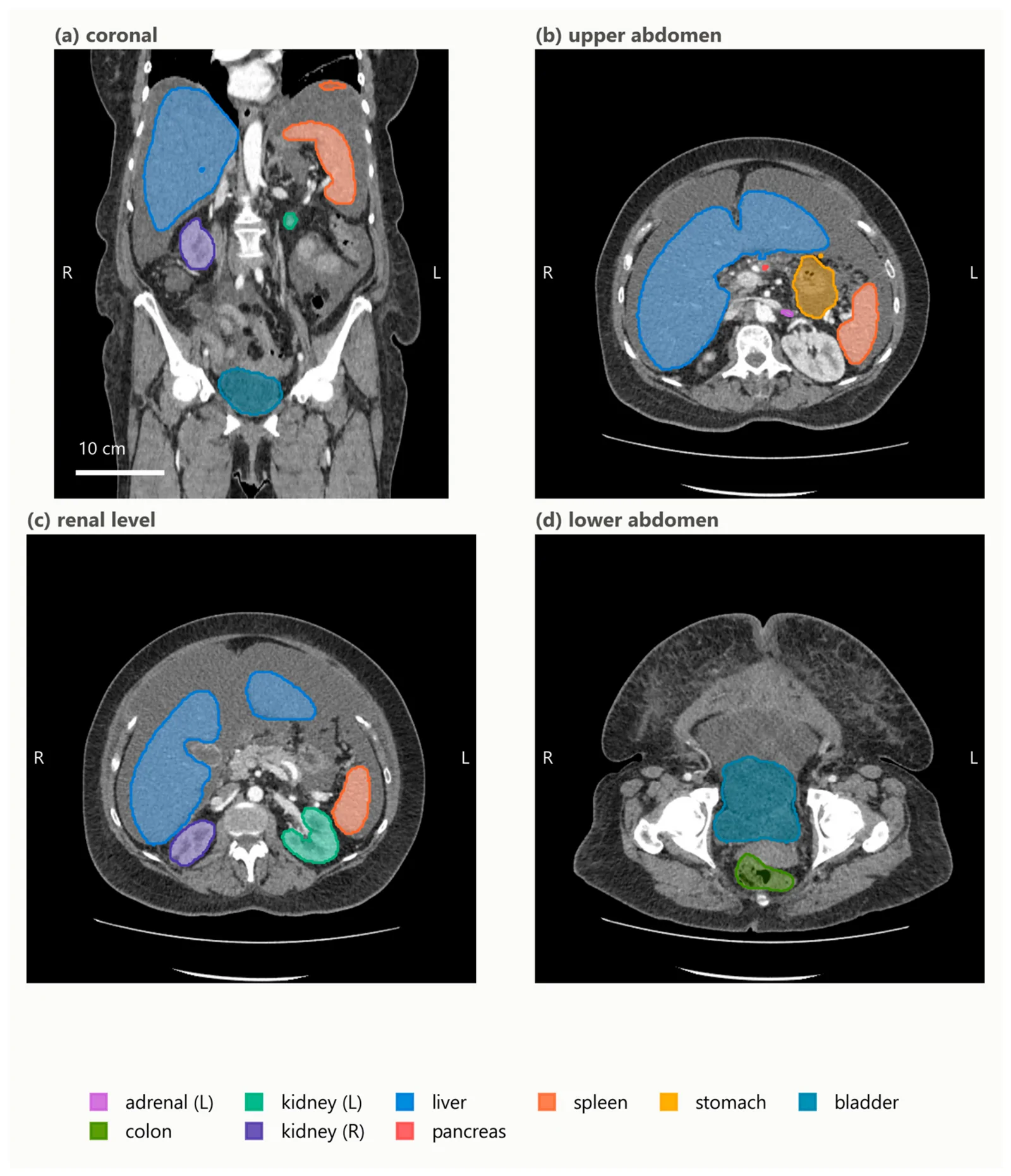
The representative TotalSegmentator output from one abdominal CT series used in the analysis. (**a**) The coronal reformat with multi-organ overlays. (**b**–**d**) Axial levels through the upper abdomen, the renal level, and the lower abdomen. Masks were generated with TotalSegmentator v2.17 using the 1.5 mm full-resolution total task and returned to the native DICOM-derived image grid; overlays are translucent so that the anatomy they are drawn against remains visible and the key lists of the structures actually shown. The same acquisition appears in Figure 3. Images are displayed in radiological convention, with the patient’s left on the viewer’s right. Only de-identified imaging from the Cancer Imaging Archive is shown.

The patient outline, used for the water-equivalent diameter, was taken from a deterministic threshold contour following AAPM Report 220 [15]. Figure 2 illustrates the segmentation output and its correspondence with the CT anatomy in the representative acquisition used for the end-to-end example.

### 2.6. Segmentation Quality Control

No mask was manually corrected, and none was used without being checked. Two procedures were applied to every series, both automated in the first instance and one of them followed by direct inspection of the images.

The first was a set of anatomical assertions run over the completed record of all 40 series. They tested properties that a correct segmentation could not violate and an incorrect one violated conspicuously: that the left-sided organ of each pair lies on the patient’s left; that the adrenal gland lies superior to the kidney on the same side; that each organ’s attenuation-derived mass falls inside a plausibility band around its ICRP 89 reference value [10]; and that the organ modulation weights vary between organs and bracket the scan mean, which they must if the weighting was being applied at all to a modulated acquisition. The first two were tripwires for the failure modes that left no other trace—a mirrored volume or an inverted head–foot ordering—because both produced masks whose volumes and Hounsfield statistics looked entirely ordinary.

Fourteen of the forty series raised at least one flag, twenty-eight flags in all. None was a laterality failure, and none was an inversion. The flags divided into four kinds. Ten were the weights failing to bracket the scan mean, which the check itself reports as expected when the acquisition extends beyond the abdomen: in a chest–abdomen–pelvis series, the scan mean includes regions no abdominal organ occupies, and the abdominal weights then sit to one side of it. Fifteen were organ masses outside the plausibility band. Two were the organ weights not varying between organs, which occurs when the tube current is effectively constant over the abdominal extent rather than when the weighting has failed; those series are separately excluded from the quantitative analysis by the acquisition-constancy criterion of Section 2.10. One was a borderline superior–inferior ordering, an adrenal and kidney centroid separated by five voxels on a series where the two structures abut.

The second procedure addressed the mass flags because an organ mass far from its reference value has two explanations that are indistinguishable in a table: a patient whose organ really is that size, and a mask that has leaked into neighbouring tissue. Only the image separates them. The organs furthest from their reference mass were rendered as contours over their own CT at three levels each, with laterality and slice index annotated on every panel, and inspected. The most extreme case in the cohort—a spleen of 671 g, 4.5 times the ICRP 89 reference—was a clean segmentation of a genuinely enlarged spleen in a renal-carcinoma patient, the contour following the organ boundary at every level over homogeneous parenchyma. The cohort was drawn from oncological collections, in which organ enlargement is common; a plausibility band built on reference values for a healthy adult was therefore expected to flag real anatomy and did.

The opposite tail did not admit that explanation. The smallest mass in the cohort was a left kidney of 4.3 g on a Philips Brilliance 64 series, segmented at 4.0 cm^3^ and not truncated by the scan boundary. No adult kidney is that size, so this is a failed mask rather than unusual anatomy: the mass plausibility check flagged it, and it was the one segmentation failure the cohort contained. It is reported here and retained in the analysis rather than removed because a cohort with its failures deleted cannot be audited. Its effect was small and is stated so that the reader does not have to take that on trust: excluding it moved the published median left-kidney modulation weight from 1.036 to 1.035. It is annotated in the mass comparison of Section 3.5.

That case also marked the boundary of what these checks can do. They are automated assertions about laterality, ordering, and mass, applied without a reference segmentation, because none exists for this cohort—the images are public and de-identified, and no manually corrected masks accompany them. Checks of that kind detect gross failure reliably: a mirrored volume, an inverted ordering, a mask that has collapsed or leaked far enough to move the organ’s mass outside a wide band. They cannot detect a mask that is systematically displaced yet plausible—a boundary drawn a few millimetres into neighbouring tissue throughout, which leaves laterality, ordering and mass all within range. Quantifying that residual error would require a manually corrected reference standard, which this study did not have and does not claim.

Segmentation error enters the index through one channel only, and it is not the channel intuition suggests. The weight is the mean tube current over the organ’s longitudinal extent, relative to the scan mean, so an error that moves the superior or inferior boundary of an organ changes which slices contribute and moves the weight. An error in the in-plane boundary at unchanged longitudinal extent does not: the same slices are averaged, with the same tube current on each. The quantity is therefore insensitive to the kind of boundary error that dominates segmentation metrics such as the Dice coefficient, and sensitive to a kind those metrics weight lightly. This is stated as a property of the construction rather than as a measured sensitivity, which the present cohort—with no manually corrected reference—cannot supply.

### 2.7. Attenuation-Derived Estimated Organ Mass

Hounsfield units were converted to mass density by piecewise-linear interpolation through reference tissue anchor points, taking the densities from ICRU Report 44 [16] and the construction from Schneider et al. [17]. Estimated organ mass is the sum of local density over mask voxels multiplied by the voxel volume.

These are **model-based estimates, not measurements**. Contrast enhancement, tube voltage, reconstruction kernel, and scanner-specific HU calibration may all affect attenuation-derived density estimates, and none was controlled in this archive cohort: contrast phase in particular varies between and within collections. The reported masses should therefore be interpreted as model-based estimates rather than physical ground truth. The HU-to-density curve itself is replaceable and travels into the provenance of every estimate; abdominal soft tissue is relatively insensitive to the choice since perturbing the water-to-muscle slope by 10% changes an abdominal organ-mass estimate by less than 1%.

### 2.8. The Anatomy-Weighted CTDIvol Index

Let an organ o occupy slices z with per-slice voxel counts $n_{o}(z)$, and let the series carry per-slice tube current I(z) over its N images. The numerator of the organ-specific modulation weight is the voxel-weighted mean tube current over the organ’s own longitudinal extent:

$${\bar{I}}_{o}=\frac{\sum_{z}n_{o}(z)\;I(z)}{\sum_{z}n_{o}(z)},$$

which weights each slice by how much of the organ it contains, so a slice through the widest part of the liver counts for more than one clipping its dome. The denominator is the mean over the whole series:

$$\bar{I}=\frac{1}{N}\sum_{z}I(z),$$

and the weight and the index are

$$w_{o}=\frac{{\bar{I}}_{o}}{\bar{I}},\;\;{CTDIvol}_{o}=w_{o}\cdot CTDIvol.$$

The weight is dimensionless and is the transferable quantity: it expresses the recorded longitudinal tube-current conditions over the organ relative to the scan mean, independently of the scanner’s own output. A weight of unity means the organ lay where the tube current happened to equal the scan average; the departure from unity is what a single whole-scan value cannot carry.

The weighting assumes that within each series, tube voltage, rotation, or exposure time, pitch and beam collimation remain fixed so that longitudinal changes in scanner output are proportional to the recorded tube current.

### 2.9. The Acquisition-Constancy Criterion

We formalised this assumption as a rule-based eligibility criterion and applied it mechanically:

A series is eligible for quantitative anatomy-weighted CTDIvol analysis only when the acquisition parameters required for scanner output to remain proportional to the recorded tube current are constant within that series to the extent verifiable from the archived DICOM headers.

Every slice header of every series was read and each output-governing attribute—tube voltage, exposure time, rotation time, pitch, and total collimation width—classified into one of four states: *Verified constant*: one value throughout. *Absent*: never written to the archived headers, so constancy can be neither confirmed nor refuted; absence alone does not disqualify a series since excluding on it would remove series for a property of the de-identified export rather than of the acquisition. *Negligible variation*: varying by less than a relative tolerance of 0.02, attributable to the numeric representation; exposure time is written as an integer number of milliseconds, so a one-unit step on a value of a few hundred is a rounding artefact. *Materially variable*: varying by at least that tolerance, which disqualifies the series.

The value of 2% follows from those two scales rather than from the data. Below it lies the following representation: at the 400–700 ms exposure times these acquisitions use, the integer millisecond step alone moves a value by up to about 0.25%, and 2% sits an order of magnitude above that. Above it lies the smallest change in technique that can actually occur since rotation time is switched in discrete steps, and the smallest of those halves or doubles it—a change of 100%. The threshold therefore separates two regimes that are two orders of magnitude apart and is not fitted to these data: any value between roughly 1% and 50% classifies this cohort identically because the only material variation observed is a factor of two.

Tube voltage was verified constant in all 40 series, as were Image Type and convolution kernel, so no series mixes acquisition or reconstruction types. Exposure time was verified constant in 35 series, negligibly variable in 3, materially variable in 1, and absent in 1; rotation time verified constant in 20, materially variable in 1, and absent in 19. Pitch verified constant in 32 and absent in 8; total collimation width verified constant in 31 and absent in 9. The full record was shipped as results/acquisition_constancy.json and the eligibility decision for every series in results/analysis_1.5mm.json.

Series failing the criterion were retained for the archive-availability, segmentation, estimated-mass, and provenance analyses and excluded only from quantitative modulation-weight and anatomy-weighted-index summaries.

CTDIvol was taken from the image header (0018, 9345) where present. Where absent, it was reconstructed from acquisition physics against an openly licensed normalised-CTDI database [18]; recorded and reconstructed values were never merged, and each series recorded that which it carried. A recorded value outside the physically possible range was treated as a corrupt attribute and fell through to reconstruction—one series recorded CTDIvol as −3.7 × 10^19^ mGy.

An organ whose mask reached the first or last slice of the series continued beyond the scan; its estimated mass was that of the scanned part, and its weight described only the exposed part. Such organs were flagged and excluded from whole-organ comparisons.

### 2.10. Organ Record Flow

Forty series and twelve requested organs gave 480 organ–series combinations. Records were produced for 455. The remaining 25 were organs that lay outside the scanned longitudinal range, so their masks were empty, and no record existed: urinary bladder in 10 series, gallbladder in 8, and seven further organs in a single 41-slice pelvic acquisition that does not reach the upper abdomen.

Two further conditions applied to the 455 records, and they were **independent axes rather than nested subsets**. Truncation is a property of the organ: 408 records were untruncated, and 47 reached a scan boundary. Index availability is a property of the series: before application of the acquisition-constancy criterion, 386 records from 34 series had a recorded or reconstructed CTDIvol and were computationally capable of carrying an index, while the remaining 69 records, from 6 series, carried a modulation weight but no index because those series had no CTDIvol by either route. The two conditions held together for 345 records; 41 truncated records still carried an index, and 63 untruncated records did not.

After exclusion of the one materially variable series, 375 records from 33 series were eligible for the quantitative anatomy-weighted-index analysis; the 11 excluded records belonged to that series. The external reference-mass comparison, which did not depend on the modulation weighting, used the 177 untruncated records of the five solid organs across the whole cohort. The full flow was in the shipped results/analysis_1.5mm.json.

### 2.11. What the Index Does and Does Not Represent

The anatomy-weighted CTDIvol index described organ-specific longitudinal tube-current modulation relative to the whole-scan CTDIvol. It did not account for scattered radiation; irradiation originating outside the organ’s segmented longitudinal extent; angular (in-plane) tube-current modulation; organ depth, position, or attenuation; patient-specific Monte Carlo radiation transport; or absorbed organ dose in milligray. It is therefore not a surrogate for absorbed organ dose and must not be read as one. What it does provide is a dimensionless, patient-specific, organ-specific measure of longitudinal modulation and a derived index in the units of the parent CTDIvol.

### 2.12. Verification and Reproducibility

Every series was screened against facts of gross anatomy that held for any adult: The left kidney lies to the patient’s left of the right kidney and the spleen to the left of the liver; the liver lies superior to the bladder and the adrenal glands superior to the kidneys. Solid-organ mass estimates fall within a wide band of reference values, and the organ weights vary within a series. These screens exist because the failures they catch leave no other trace.

Analyses were run with Python 3.14 (the package supports 3.10 and later), TotalSegmentator v2.17 (total task, 1.5 mm full-resolution model) on PyTorch 2.11 with CUDA 12.8, pydicom 3.0, and NumPy 2.5, using an NVIDIA RTX 3080 (NVIDIA Corporation, Santa Clara, CA, USA). Every reported value was re-derived from the per-series records by the test suite, including regenerating the complete analysis table and comparing it. The pipeline was organised in four layers—acquisition, organ record, analysis, and figures—each regenerated by a single command and each writing a machine-readable record that the next layer read; the acquisition–parameter check of Section 2.9 was a fifth, run over the completed records. Figure 1 gives the stages, the count surviving each, and the verification step attached to each. The repository, its release tag, commit hash, and archived version DOI are given in the Data Availability Statement, and the command for each layer is in its README rather than here.

### 2.13. Use of Generative Artificial Intelligence

A generative artificial intelligence assistant (Claude Opus 5, Anthropic) was used as a tool in developing the software described in Section 2.1, Section 2.2, Section 2.3, Section 2.4, Section 2.5, Section 2.6, Section 2.7, Section 2.8, Section 2.9, Section 2.10, Section 2.11 and Section 2.12 and in drafting and editing the text of this manuscript. It was not used to generate, impute, or select any reported value. Every number in this article was produced by executable code in the cited repository, was re-derived from the per-series records by the automated test suite described above, and was verified by the author against the underlying records. The study design; the eligibility rules; the quality-control criteria; and all scientific judgements, interpretations, and conclusions are the author’s, who takes full responsibility for the content of this article.

## 3. Results

### 3.1. Cohort and Records

Forty series were analysed—ten from each of GE HealthCare, Chicago, IL, USA, Siemens Healthineers, Erlangen, Germany, Canon Medical Systems, Otawara, Tochigi, Japan and Philips Healthcare, Best, The Netherlands—drawn from 21 collections and 23 scanner models. Of 480 organ–series combinations, 455 organ records were produced across 12 organs, with the flow as given in Section 2.10. Figure 2 shows the segmentation output for a representative acquisition.

Of the 40 segmented series, 39 met the acquisition-constancy criterion of Section 2.9. One archived GE series contained two blocks with different exposure and rotation times; it was retained for the segmentation, estimated-mass, and archive-availability analyses and excluded from the modulation-weight and anatomy-weighted-index summaries. The quantitative modulation analysis therefore rests on 375 organ records from the 33 eligible series that also carry a CTDIvol.

### 3.2. Organ-Specific Modulation Weights

Across the eligible series, organ-specific modulation weights span 0.59 to 1.69. The anatomy-weighted index therefore departs from the whole-scan CTDIvol by up to roughly 70% in either direction within a single acquisition, which is the variation the index exists to express.

**Figure 3:**
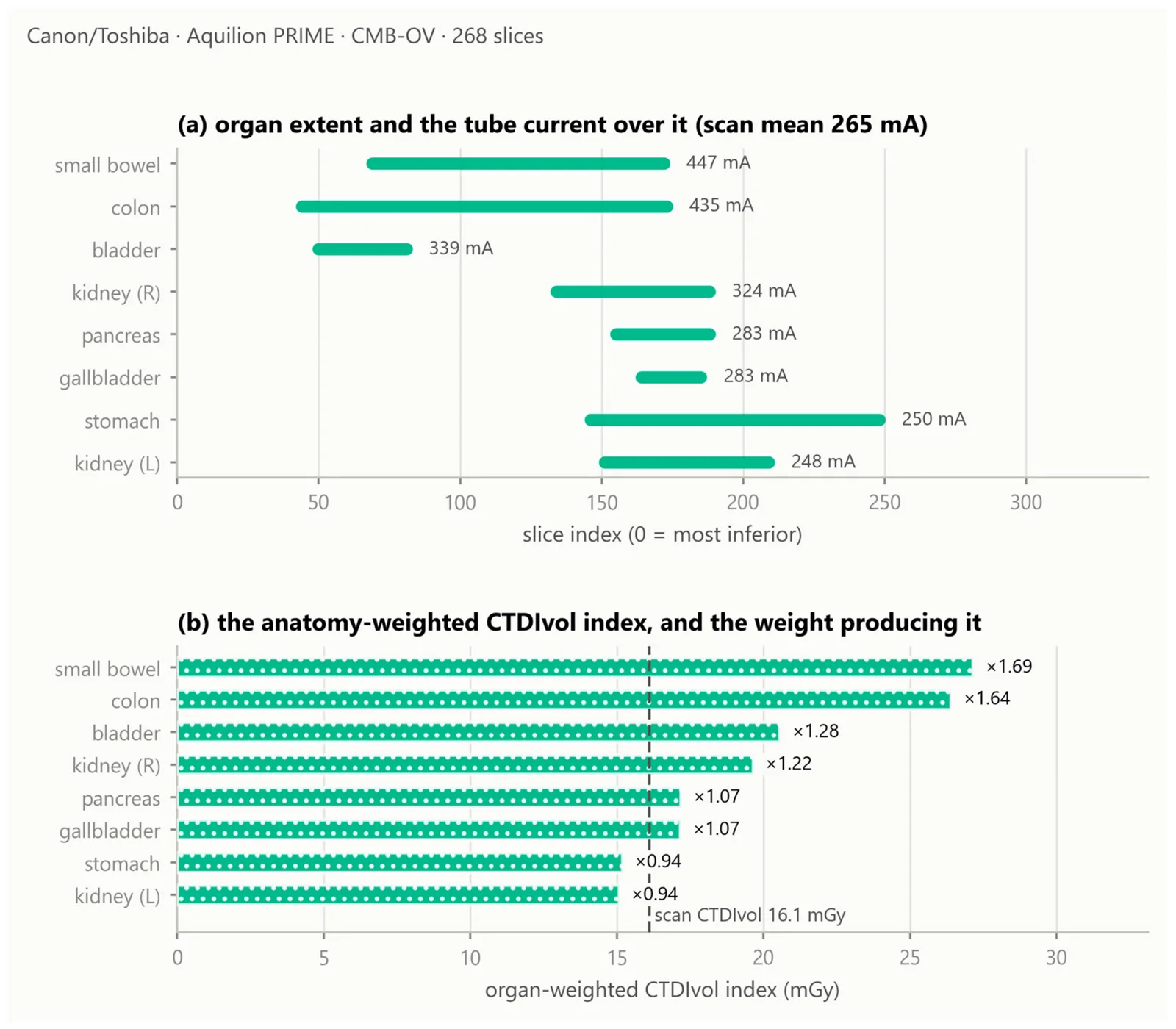
One acquisition end to end. (**a**) Each organ’s longitudinal extent, annotated with the mean tube current recorded over it, against a scan mean of 265 mA. (**b**) The resulting anatomy-weighted CTDIvol index, with the organ-specific modulation weight beside each bar; the dashed line is the whole-scan CTDIvol of 16.1 mGy. The bars are modulation-weighted indices, not absorbed doses.

Figure 3 shows one acquisition end to end: a Canon/Toshiba Aquilion PRIME series of 268 slices with a recorded CTDIvol of 16.1 mGy and a scan mean tube current of 265 mA. The small bowel and colon, lying in the inferior abdomen where the modulation raised the current to about 447 and 435 mA, take weights of 1.69 and 1.64, respectively, giving indices near 27 mGy; the left kidney and stomach, higher in the scan, take weights of 0.94 and indices near 15 mGy. Two organs in the same acquisition thus differ by a factor of 1.8 in their anatomy-weighted index, a difference no whole-scan value can express.

### 3.3. Availability of a Dose Index in the Archived Headers

**Figure 4:**
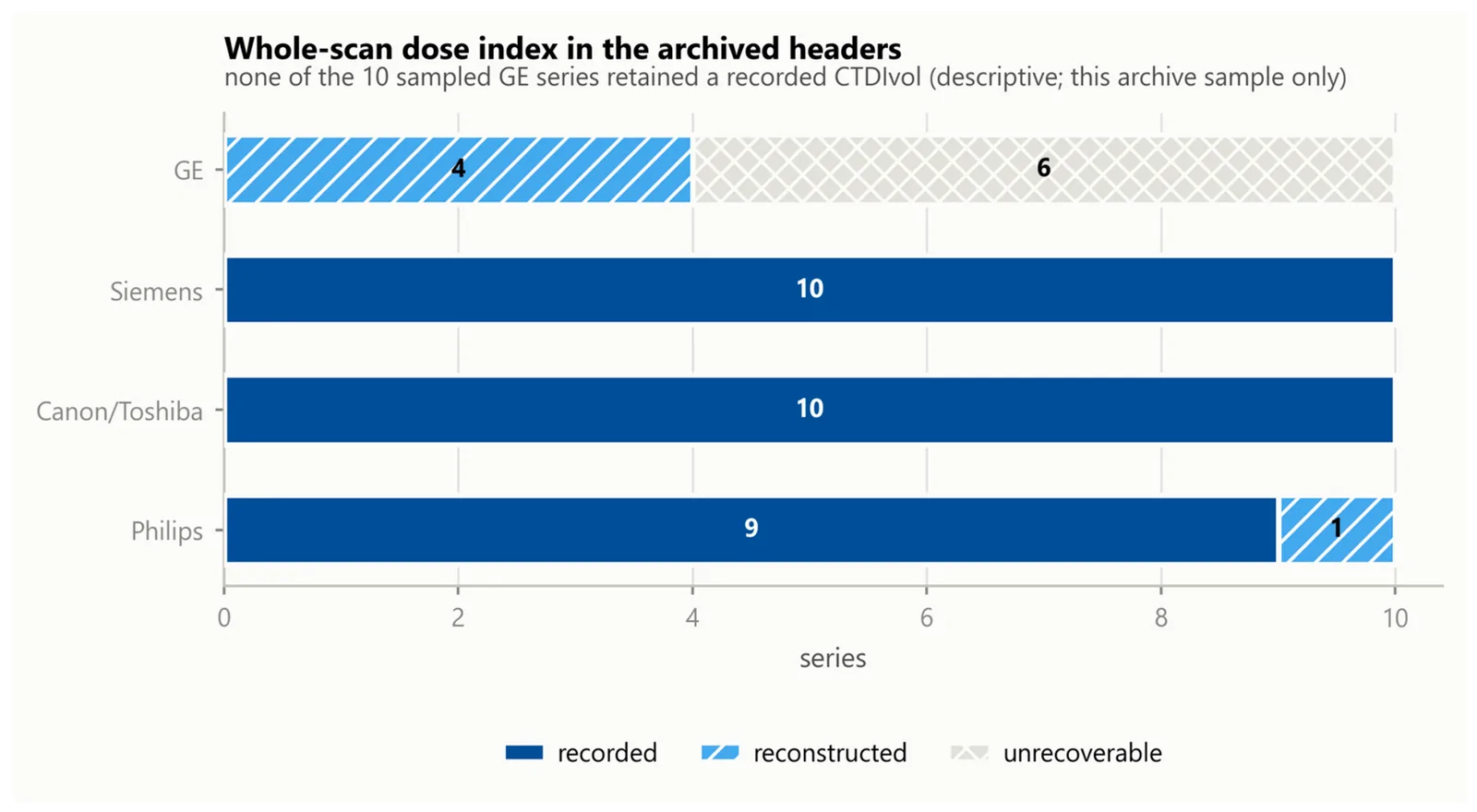
The availability of a whole-scan dose index in the archived DICOM headers, by manufacturer, in this TCIA sample. A series counted unrecoverable when it retained no CTDIvol in its header, and its scanner lay outside the open coefficient database. Segments are distinguished by fill pattern as well as tone.

Across the cohort, 29 of 40 series retained a recorded CTDIvol in the archived DICOM headers, 5 were reconstructable from acquisition physics, and 6 were neither (Figure 4).

All six unrecoverable series were GE, and none of the ten sampled GE series retained a recorded CTDIvol in the archived headers; the other three manufacturers retained 1 in 29 of 30. Retention of the attributes on which dose monitoring depends is itself a reported problem: monitoring built on the DICOM structured report is limited by what an installation writes and keeps [19], and compliance with dose-reporting requirements has been found incomplete even where they are mandated [20]. These counts are reported descriptively. No significance test is applied: series drawn from a curated archive are not independent with respect to collection, contributing site, scanner model, export pathway, or de-identification, and a *p*-value computed over that structure would describe a sampling model the data do not satisfy. For the six unrecoverable series the organ masks, volumes, mass estimates, and modulation weights are all computable and reported, but no anatomy-weighted index exists for them.

### 3.4. How Far the Reconstructed CTDIvol Agrees with a Recorded One

Where the header retains no CTDIvol, the value is rebuilt from acquisition physics and an open coefficient table [13], and the two origins are kept apart in every record because the uncertainty they carry differs. That difference has to be quantified rather than asserted.

It cannot be quantified in the way one would first attempt. No series in this cohort carries both values: the reconstruction runs only where the header has none, so the recorded and reconstructed populations are disjoint by construction. The comparison was therefore made by forcing the reconstruction on the series that do carry a recorded value, where the recorded value plays no part in producing the reconstructed one.

Of the 29 series with a recorded CTDIvol, 8 could be reconstructed; the remainder are on scanner models absent from the open table. Across those eight, the median absolute difference is 10.5%, but the differences do not form a spread. Five agree to within 12%—two Aquilion PRIME at −8.8%, one Aquilion ONE at −8.3%, and two iCT 256 at a median −2.0%—and three do not, two Aquilion Prime SP at a median +58.0% and one SOMATOM Definition Flash at +84.0%. The disagreement is consistent within a scanner model rather than scattered across series, which locates it in the tabulated coefficient for those models and not in the per-series inputs; model resolution is exact after normalisation and rejects near misses, and the spiral pitch, checked on all eight, is recorded in every case.

The limitation of this measurement is more important than its result. A model can be checked here only if some series in the cohort retained a recorded CTDIvol for it, and no GE series retained one. Of the five series whose index rests on a reconstructed CTDIvol, one is a Philips iCT 256, a model measured above at a median −2.0%; the other four are GE, on models the comparison cannot reach. The reconstruction is therefore unverified precisely where this study leans on it hardest, and that is a property of what the archive kept rather than of the method. Results resting on a reconstructed value are marked as such throughout.

### 3.5. External Reference Comparison of Estimated Organ Mass

**Figure 5:**
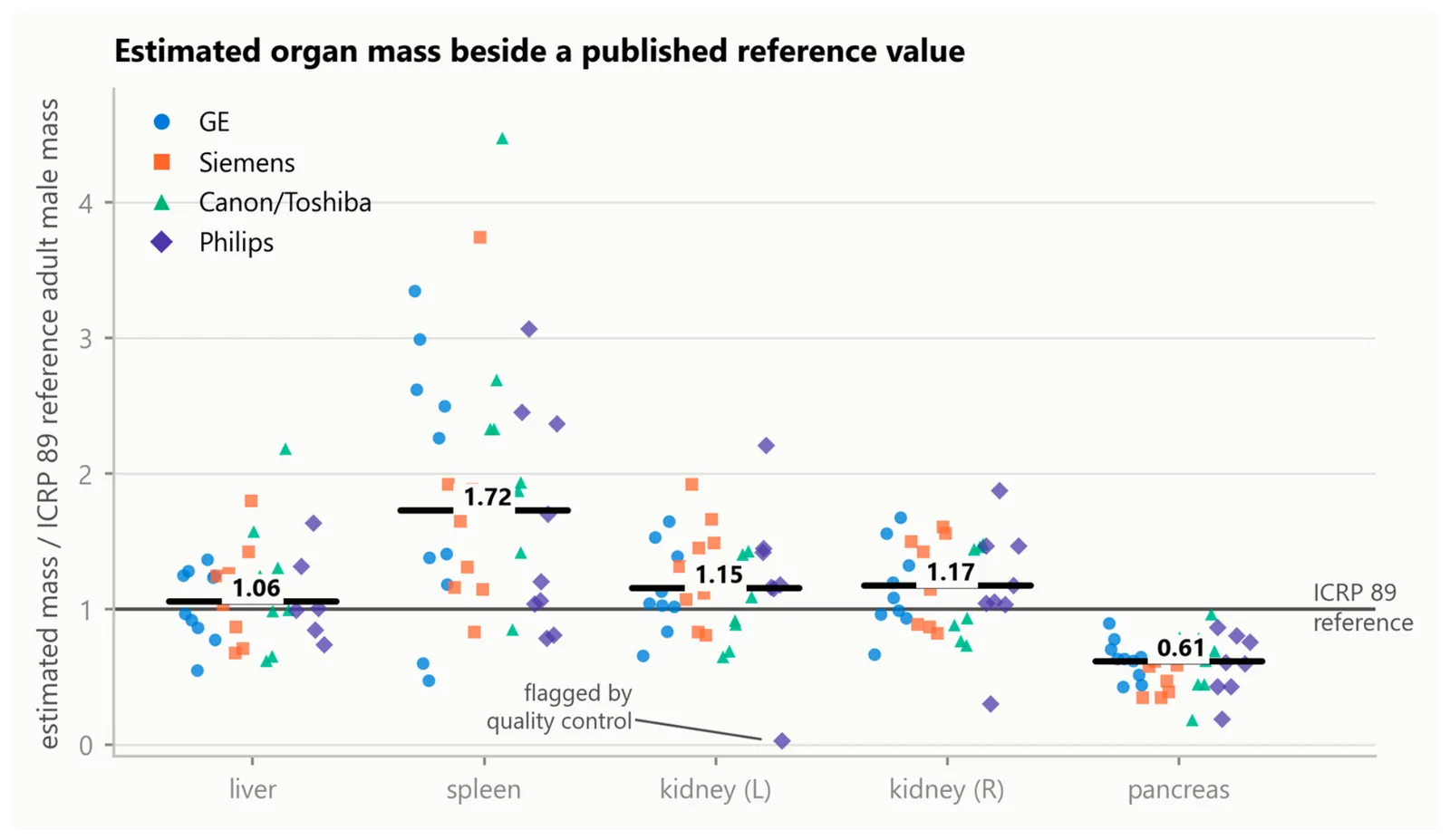
The attenuation-derived estimated organ mass relative to the ICRP 89 reference adult male mass, by manufacturer. Each marker is one organ in one series; the horizontal bar is the median across all manufacturers. Organs truncated by the scan boundary are excluded. The manufacturer is encoded by marker shape as well as colour, so the figure is readable in greyscale. The annotated point is the one mask the quality control of Section 2.6 flagged as a segmentation failure, retained here rather than removed.

Table 1 and Figure 5 place attenuation-derived estimated organ mass beside the ICRP 89 reference adult male values [10] over the 177 untruncated records of the five solid organs.

Estimates for the liver were broadly consistent with the reference value, within 6%, and the kidneys within 17%. Two organs departed more substantially: the pancreas estimate was 39% below the reference and the spleen 72% above. Neither was adjusted; possible explanations are examined in Section 4.

### 3.6. What Limits an Organ-Level Modulation Analysis

**Figure 6:**
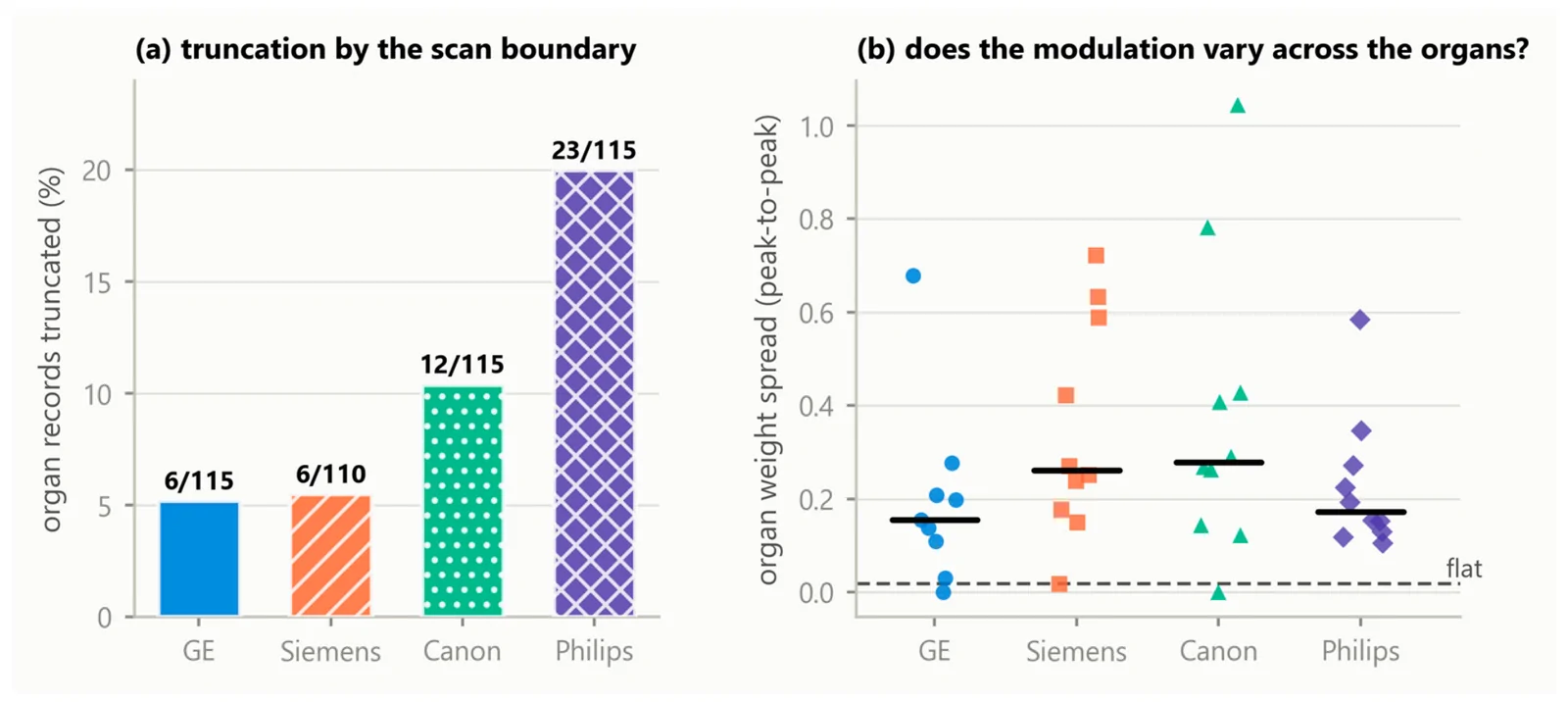
What limits an organ-level modulation analysis. (**a**) Percentage of organ records truncated by the scan boundary, by manufacturer, annotated with the counts, over all 40 segmented series. (**b**) Peak-to-peak spread of the organ-specific modulation weights within each of the 39 series eligible for quantitative modulation analysis; the dashed line is the threshold below which a series carries no usable variation.

Two conditions reduce what such an analysis can measure, and both differ across the sampled manufacturers (Figure 6).

Truncation by the scan boundary affected 5.2% of organ records on GE, 5.5% on Siemens, 10.4% on Canon/Toshiba, and 20.0% on Philips, reflecting the scan ranges of the sampled acquisitions rather than any property of the scanners. The organs most often cut are the colon and small bowel.

Three of the thirty-nine series eligible for quantitative modulation analysis showed a peak-to-peak spread of organ weights below 0.02: their tube current does not vary across the abdominal organs, so the weighting has nothing to express. These series passed the modulation screen at selection, where the current varies across the whole scan; the flatness is local to the abdomen. They are uninformative for this analysis rather than faulty.

## 4. Discussion

**Principal finding.** Organ-specific modulation weights span 0.59 to 1.69 across the eligible cohort, and within a single acquisition, two organs differed by a factor of 1.8 in their anatomy-weighted index. Longitudinal modulation therefore produces organ-specific exposure conditions that a single whole-scan CTDIvol cannot represent, and the magnitude is large enough to matter for any organ-level analysis built on that value.

The direction of that finding is the part likely to hold; the size of it is not. A weight span is a property of the protocols, patient habitus, and modulation settings that happen to be present, and these 39 series were assembled from oncological collections by a vendor-balanced quota rather than sampled from any clinical population. A cohort with different body sizes, a different mix of examination types, or different modulation strength would produce a different span. What the numbers here establish is that the departure is not small and cannot be assumed away; they do not establish how large it is in any particular clinic, and the same caution applies to the availability fractions below.

**Interpretation.** The weight is a direct, dimensionless summary of how the recorded tube current was distributed over an organ’s own longitudinal extent in that patient. It requires no phantom, no simulation, and no additional acquisition—only metadata the scanner already writes and a segmentation obtained at inference.

**Relation to previous work.** The quantity is that of Khatonabadi et al. [3] and Tian et al. [4], who established the organ-specific weighted CTDIvol and validated it against Monte Carlo organ dose; this study did not invent it and adds nothing to those validations. What differs is the setting. That line of work, and the modulation dosimetry around it, proceeded from single-institution cohorts with one or two scanner models and manual or semi-automatic contours, drawing in part on raw projection data or vendor-supplied modulation profiles that archived DICOM does not retain. Here, the same quantity is obtained from archived headers alone across four manufacturers and 23 scanner models, with contours produced automatically.

Three recent studies sit closest and differ in endpoint rather than in quality. Li et al. [5] characterise SSDE(z), a patient-size-adjusted dose index evaluated at each longitudinal position; it is related but not the same quantity since the weighting here is by segmented organ occupancy of the tube-current profile rather than by patient size at a given position. Nuntue et al. [6] also derive modulation profiles from DICOM headers and go further than this work in estimating absorbed organ dose with Monte Carlo simulation and measurement validation; this study deliberately stops short of absorbed dose and addresses instead archive feasibility, multi-vendor availability, quality control, and an open implementation. Eom et al. [7] likewise use TotalSegmentator for automated dose calculation, but their endpoint is the effective dose from body regions and DLP conversion factors, whereas the present work uses individual organ masks, the per-slice tube current, and an organ-specific longitudinal weighting.

The contribution is therefore not a new dose index or an improvement on the published Monte Carlo validations. It is an open, multi-vendor operationalisation and empirical characterisation of the organ-weighting layer under the constraints of real archived DICOM.

**What the index is and what it is not.** As set out in Section 2.11, the index addresses longitudinal modulation alone. It does not account for scatter, for irradiation originating outside the organ’s segmented extent, for angular modulation, for organ depth and attenuation, or for radiation transport, and it is not an estimate of absorbed organ dose in milligray. Its value lies in isolating one well-defined contribution—the longitudinal one—and reporting it patient-specifically and reproducibly.

**External reference comparison of estimated mass.** Liver and kidney estimates were broadly consistent with ICRP 89 reference values, which is the expected behaviour if the segmentation and the density model are working. The pancreas estimate sits 39% below the reference. The pancreas is the weakest of these organs in TotalSegmentator’s own validation (Dice 0.887, against 0.965 for the liver, 0.983 for the spleen, and 0.953 and 0.939 for the kidneys [8]), which supports reduced boundary agreement, but Dice is symmetric and does not establish the direction of a disagreement, so it does not by itself demonstrate under-segmentation. The contrast phase, reconstruction kernel, and genuine anatomical variation in this cohort are alternative contributors that the present design cannot separate. The source of the discrepancy cannot be determined without subject-level reference contours or clinical ground truth.

The spleen estimate sits 72% above the reference. The four largest cases—4.47, 3.74, 3.34, and 3.06 times the reference value—were reviewed slice by slice against their own images: each contour follows the splenic boundary with correct laterality; tracks the notch at the hilum; shows no leakage into liver, kidney, or stomach; and forms a single connected component, so no accessory spleen was included. Their mean densities, 1.052 to 1.078 g/cm^3^, are unremarkable for splenic tissue, so the elevation arises from segmented volume rather than from the density model, and the spleen is the best-segmented of these organs in the segmenter’s validation. The cases arise on four different manufacturers. The cohort is oncological—renal cell, colorectal, and adrenal carcinoma—in which splenomegaly is common, and the ICRP reference adult is not of that population. The observed elevation was most consistent with cohort anatomy among the explanations examined, although subject-level pathological confirmation was unavailable.

**Availability in archived headers and its confounders.** In this sample, the availability of a recorded CTDIvol differed markedly with manufacturer. This is an observation about archived DICOM headers in one curated archive, not a statement about scanner implementations. The present design cannot distinguish between scanner implementation, scanner generation, acquisition site, DICOM export pathway, PACS processing, de-identification, archive curation, and collection composition as the origin of a missing attribute; several of these are confounded with manufacturer through collection membership. The practical consequence stands regardless of cause: a retrospective organ-level analysis drawn from an archive will lose a manufacturer-associated fraction of its cohort before segmentation is considered, and a study that does not report which series were lost will under-represent that manufacturer silently.

**What the reconstructed values do to the index and what they do not.** Section 3.4 measures how far a reconstructed CTDIvol departs from a recorded one; the question that follows is what such a departure does to the index built on it, and the two halves of the answer are of different kinds. The first is structural. The modulation weight is a ratio of tube currents, as defined in Section 2.8, and CTDIvol does not enter it; the index is that weight multiplied by CTDIvol. An error in CTDIvol therefore appears in the index at 1:1 and cannot reach the weight at all, so the modulation results of Section 3.2 are independent of it by construction rather than by measurement. The second half is empirical and concerns how much of the cohort is exposed. Of the 33 series carrying an index, 4 rest on a reconstructed CTDIvol, covering 44 of 334 organ records. Recomputing every table over the series with a recorded value alone moves the per-organ median modulation weight by at most 0.048 and the per-organ median anatomy-weighted CTDIvol by at most 1.11 mGy. The conclusions of this study do not depend on those four series, but the reason for saying so is not that they can be discarded. Removing them removes GE from the weighted tables entirely, from three series to none, because no GE series in this cohort retained a recorded CTDIvol at all. The multi-vendor reach of the modulation results rests on values whose accuracy Section 3.4 could not check, and that is the honest statement of where this study is weakest.

**Why acquisition constancy has to be screened.** The acquisition-constancy criterion identified one series in which tube current alone was not proportional to scanner output because the acquisition changed rotation and exposure time part-way through. Excluding it prevents a mixed acquisition from entering the quantitative modulation analysis and illustrates why constancy must be verified rather than assumed: nothing in the images, the segmentation, or the weights themselves would have revealed it. A current–time product would be the more faithful weighting in general; it is not adopted here because exposure time is absent from the archived headers of one other series and could not be applied uniformly across the cohort.

**Reproducibility and open implementation.** The implementation is MIT-licensed; no imaging is redistributed, and every reported value is re-derived from the per-series records by an automated test suite that regenerates the analysis tables and compares them. The acquisition procedure, the analysis, and the figures are each a single command. The components differ in status and are not claimed as uniformly open: the imaging is publicly accessible under collection-specific licences; the segmentation software and the total task weights are openly redistributable; the normalised-CTDI database is CC BY. The HU-to-density anchor values are used by citation to ICRU Report 44 [16] and Schneider et al. [17] rather than redistributed, and no Monte Carlo coefficient table is included at all.

**The boundary to absorbed organ dose.** Converting an anatomy-weighted index into an absorbed organ dose requires CTDIvol-normalised coefficients with a patient-size correction of the kind applied over patient model libraries by Tian et al. [4], together with the transport considerations listed in Section 2.11. Those coefficient sets are published in subscription journals or distributed with research software under terms granting use but not redistribution, which reflects a publishing convention for Monte Carlo reference data rather than any deficiency in the coefficients themselves. Normalised-CTDI data of the kind used here to reconstruct a missing whole-scan index has been published under CC BY [18], which shows the convention is movable. The software accordingly refuses to emit a dose in milligray unless supplied with a coefficient table carrying its citation, DOI, licence, and source hash.

**Limitations.** Ten series per manufacturer supports a median and an interquartile range, not a distributional claim, and series within the archive are not independent with respect to collection, site, scanner model, or export pathway. The design is balanced by manufacturer and not by scanner model, and the two are not interchangeable: those ten series per manufacturer are spread over nine distinct GE models, six Siemens, four Canon/Toshiba, and four Philips, so most individual models are represented by one to three series. Anything stated here about a manufacturer is therefore a statement about a small, model-diverse sample of that manufacturer’s installed base, and nothing in this study supports a claim at the level of a particular scanner model. The cohort is oncological and not a reference population. Contrast phase, tube voltage, and reconstruction kernel were not controlled, and all affect attenuation-derived mass estimates. A single segmentation model was used, so segmentation behaviour and cohort anatomy cannot be separated. There is no subject-level ground truth for organ mass. Rotation time, pitch, and collimation are absent from the archived headers of some series (19, 8, and 9, respectively), so their constancy within those series could not be fully verified and is assumed from the single-acquisition representation; the acquisition-constancy criterion is therefore a screen against detectable violations rather than a guarantee. The index addresses longitudinal modulation only, as set out in Section 2.11, and no absorbed dose is reported.

**Future work.** Coefficients computed with an open-source Monte Carlo engine would carry no licensing constraint and would permit the transport terms this index omits; an independent segmentation model on the same series would separate segmentation behaviour from cohort anatomy for the pancreas and spleen, and a larger archive cohort would allow for the availability observation to be examined with collection and site modelled explicitly rather than confounded.

## 5. Conclusions

This study did not compute absorbed organ dose and did not propose a new index. It took an organ-specific weighted CTDIvol already reported in the literature and established what it yields when operationalised openly across manufacturers, from metadata a scanner already records and organ masks obtained at inference. Organ-specific modulation weights spanned 0.59 to 1.69, and two organs within one acquisition differed by a factor of 1.8 in their index, so the variation a single whole-scan value conceals is substantial. The approach ran across four manufacturers using publicly accessible imaging data and openly redistributable software components, and in this archive cohort, the availability of the whole-scan CTDIvol the index scales varied markedly between manufacturers, which constrains any retrospective analysis of this kind. The index is a step before conversion to absorbed organ dose, not a substitute for it: that conversion requires Monte Carlo coefficients and the transport terms this index omits. The implementation and derived records are openly available, provenance-aware, and reproducible from the shipped results.

## Figures and Tables

**Table 1:** Attenuation-derived estimated organ mass beside International Commission on Radiological Protection (ICRP) Publication 89 reference adult male values, untruncated organs only. The reference is an external anchor, not a subject-level ground truth.

Organ	*n*	Median Estimated Mass	Ratio to ICRP 89
liver	34	1901 g	1.06
spleen	38	259 g	1.72
kidney (left)	34	179 g	1.15
kidney (right)	35	182 g	1.17
pancreas	36	86 g	0.61

## Data Availability

The software supporting this study, ctsegdose-core, is openly available under the MIT licence at https://github.com/Institute-of-One/ctsegdose-core (accessed on 28 August 2026), release v0.1.2 (commit 0ca9d57ef9fbc1a321cd5b671d21eb5857def518), archived at Zenodo under 10.5281/zenodo.22143005. The machine-readable results the manuscript quotes, the per-organ records, the analysis tables, the acquisition–parameter check and the figure scripts are included in that repository, together with the mask-review overlays underlying Section 4. No DICOM imaging is redistributed. Every series analysed is identified in data/PROVENANCE.json by collection, collection DOI, Series Instance UID, manufacturer, model, licence, and retrieval date and is retrievable directly from the Cancer Imaging Archive by following docs/REPRODUCING_DATA.md. The forty series were retrieved under Creative Commons Attribution licences (33 under CC BY 4.0, 7 under CC BY 3.0) as recorded per series; the licence terms of each originating collection and the TCIA Data Usage Policy apply. Segmentation used TotalSegmentator [8]. Its software code is distributed under the Apache 2.0 licence, and the total task model weights used here are likewise stated by the project to be openly available under Apache 2.0; other tasks in that project require a separate licence and were not used. No model weights are redistributed with this work. No organ–dose coefficient table is redistributed for the licensing reasons set out in Section 4.
